# Iatrogenic Intraoperative Injury of the Hard Palate During Septoplasty: A Case Report and Literature Review

**DOI:** 10.1155/crot/8808966

**Published:** 2026-04-05

**Authors:** Islam Mourad, Nasser Alalyani, Rafed Abdulhameed, Abdulrahman Alabdulqader, Khalid Aldilaijan

**Affiliations:** ^1^ Department of Otorhinolaryngology-Head & Neck Surgery, King Fahd Military Medical Complex, Dhahran, Saudi Arabia; ^2^ Department of Otorhinolaryngology-Head & Neck Surgery, Imam Abdulrahman Bin Faisal University, Dammam, Saudi Arabia, iau.edu.sa

**Keywords:** hard palate injury, high-arched palate, septoplasty

## Abstract

A rare complication of septoplasty is an iatrogenic hard palate injury. In this report, we present a case of a hard palate injury while performing a septoplasty in a 32‐year‐old male, which was detected and managed intraoperatively. Key features of our patient include a high‐arched palate, open bite, and nasal obstruction due to a deviated nasal septum. Postoperative evaluation showed successful healing which was free from recurrences. A review of the literature for similar cases highlights the role of anatomical variations from patient to patient, detection times of the injury, and repair methods in the management of such an event. Nonetheless, it is vital for the patient to undergo thorough preoperative assessment and for the surgeon to exercise caution intraoperatively to prevent/manage this adverse event efficiently.

## 1. Introduction

Septoplasty is a commonly performed procedure to correct nasal septal deviation. Like every procedure, septoplasty is associated with potential complications. Well‐known complications include bleeding, septal hematoma, septal perforation, infection, septal abscess, nasal synechiae, anosmia/hyposmia, cerebrospinal fluid leak, and complications related to anesthesia [1]. Hard palate injury is one of the rare complications of septoplasty, with only a handful of literature available that documents such an event [2].

Hard palate injury results in a defect that may result in an oro‐nasal fistula. Patients with oro‐nasal fistula may complain of hypernasal speech, regurgitation of fluids into the nasal cavity, and compromised oral hygiene [2].

## 2. Case Report

**FIGURE 1:**
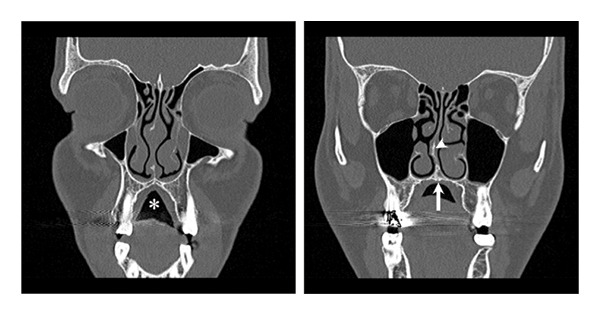
Noncontrasted CT scan of the paranasal sinuses, coronal view, showing a constricted high‐arched palate (star), wide maxillary crest which is a potential area for palate injury (arrow), deviated nasal septum (arrowhead), and hypertrophied inferior turbinates.

A 32‐year‐old male presented to the rhinology clinic complaining of nasal obstruction for several years. He was suffering from nasal obstruction predominantly on the right side. Additionally, he had a longstanding history of loud snoring, excessive daytime sleepiness, and observed apneas during sleep. He had a body mass index (BMI) of 34 kg/m^2^, a high‐arched palate, and an open bite. Anterior rhinoscopy showed a deviated nasal septum with hypertrophied inferior turbinates. Computed tomography (CT) of the paranasal sinuses was done, and it showed a deviated nasal septum, hypertrophied inferior turbinates, a wide maxillary crest, and a constricted high‐arched palate (Figure 1). A home sleep test was conducted and revealed moderate obstructive sleep apnea (OSA) with an apnea‐hypopnea index (AHI) of 20/h.

Based on that, he was advised for weight reduction along with the use of continuous positive airway pressure (CPAP); prescriptions included an intranasal corticosteroid spray and saline nasal irrigation. The patient did not tolerate the CPAP and showed no clinical improvement on nasal medications. Therefore, he was advised to seek the maxillofacial department to consider maxillary expansion and skeletal advancement surgery. The patient was counseled about orthodontic and maxillofacial plans. However, he returned to the ENT department seeking nasal surgery, given its shorter treatment plan.

**FIGURE 2:**
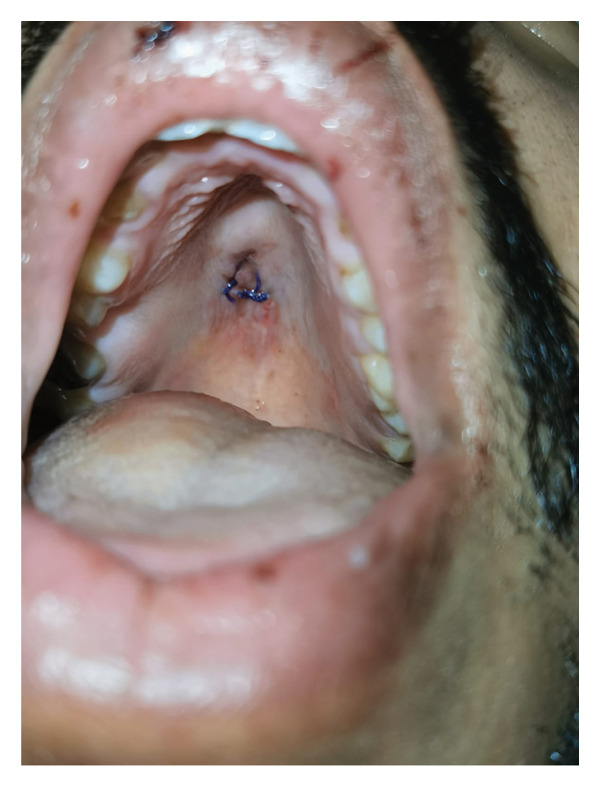
Throat examination on Day 1 postoperatively showing the repaired palatal injury.

**FIGURE 3:**
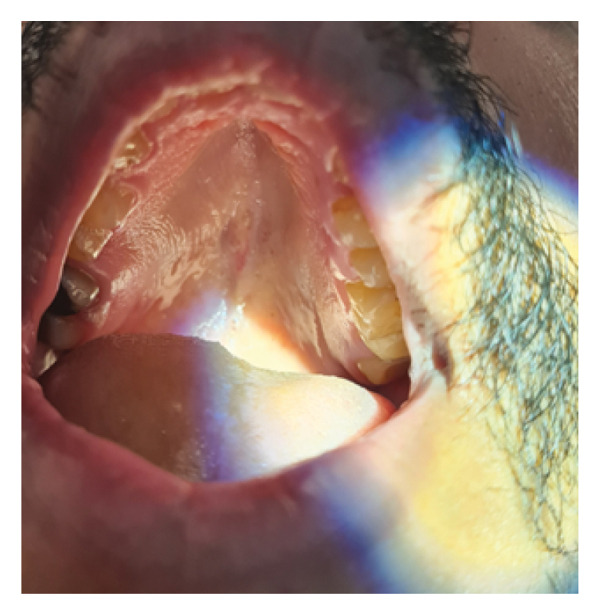
Healed palate is demonstrated during postoperative follow‐up visits. Photo is taken at 1‐year postoperatively.

He underwent septoplasty and bilateral inferior turbinoplasty. During the osteotomy of the hard maxillary crest, the surgeon noticed a sudden release of the maxillary crest (vertical fracture through the maxillary crest bone) and detachment at its floor, with irregular soft tissue below. Intraoperative oral examination during the same procedure confirmed a palatal perforation with an inverted V‐shaped cut wound of the hard palate mucosa. A primary repair was performed during the same procedure using Vicryl 3‐0 simple stitches transorally. The patient was examined on Day 1 postoperatively and discharged home (Figure 2). Postoperative follow‐up visits showed improved nasal patency and a healed palate (Figure 3).

## 3. Literature Review and Discussion

Of the published case reports, a variety of factors were present that lead to the complication of hard palate injury during septoplasty. These include the presence of a submucous cleft palate [3], high‐arched palate [4, 5], and a retained intranasal splint [6]. Our patient had a constricted (narrow) high‐arched palate with a wide maxillary crest. In our opinion, the usage of a mallet with chisel or osteotome to break the thick and hard base of the septum played a role in the occurrence of the iatrogenic injury to the hard palate.

Previous case reports about iatrogenic hard palate injury and oro‐nasal fistulae after septoplasty share some common clinical features. Patients presented with hypernasal speech [5, 7, 8] and fluid regurgitation into the nasal cavity [2, 5–9].

The literature showed variable detection and intervention time for this complication. Similar to our case, intraoperative detection with primary repair was reported [5]. Early presentation (from 3 to 14 days postoperatively) and late presentation (from 2 to 24 months postoperatively) were all described in the literature [2–4, 6–9].

The method of repair may differ from case to case. Primary repair with simple stitches can be suitable for injuries detected during septoplasty [5]. Patients who present with postoperative oro‐nasal fistula can be managed with a local mucoperiosteal flap [3, 4, 8], von Langenbeck palatoplasty [9], reduction of the hard palate bony segment [2], and a multilayer reconstruction of the fistula using Tutoplast and mucoperiosteal flap [7]. Due to the small, slit‐like nature of the injury in our patient, the repair was rather straightforward, with Vicryl 3‐0 simple stitches being placed transorally to close the defect successfully. A summary of comparison between the reported cases is explained in Table 1.

**TABLE 1:** Summary of reported cases of iatrogenic palate injury during/after septoplasty in the literature.

Reference	Proposed aetiology/mechanism	Timing of detection or presentation	Symptoms	Repair method	Outcome
Tilaveridis et al. [2]	Displaced fracture of part of the hard palate	3 days postop	Fluids regurgitation into nasal cavity	Reduction of the displaced bone of hard palate and simple suture closure of oral mucosa	Successfully healed

Erosy et al. [3]	Submucous cleft with bifid uvula	Immediately postop	Speech and voice change	Two‐layer flaps nasal mucosal flap and oral rotational mucoperiosteal flap	Previous failed first attempt of 2 year repair. Successfully healed after a second attempt.

Gökdemir and Bal [4]	High‐arched palate	6 months postop	Hypernasal voice	Multilayer repair: Mucoperiosteal flap for nasal side for oral side.	Successfully healed

Yadav et al. [5]	High‐arched palate	Intraop	Hypernasal voice and fluids regurgitation into nasal cavity	Primary transoral repair using Vicryl sutures	Failed 1 year repair. Spontaneously healed after obturator use for 3 weeks.

Sheikh and Nadeem [6]	Retained intranasal splint	2 years postop	Fluids regurgitation into nasal cavity	Palatal mucoperiosteal flap	Successfully healed

Alhedaithy and Alsaleh [7]	Aggressive resection of the maxillary and palatine crests, with possible utilization of an inferiorly directed osteotome	2 months postop	Hypernasal voice and fluids regurgitation into nasal cavity	Multilayer repair: Mucosal free flap for nasal side and tutoplast with mucoperiosteal rotational flap for oral side.	Successfully healed

Motazedian et al. [8]	Aggressive osteotomy of septal bony part	2 years after septorhinoplasty. With 2 previous failed repair attempts	Hypernasal voice and fluids regurgitation into nasal cavity	Multilayer repair: Mucosal hinge flap for nasal side and mucoperiosteal rotational flap for oral side.	Successfully healed

Kaya et al. [9]	Not mentioned	15 days postop	Fluids regurgitation into nasal cavity	von Langenbeck palatoplasty	Successfully healed

Islam et al. (our case)	High‐arched palate with wide maxillary crest	Intra‐op	Asymptomatic	Primary transoral repair using Vicryl sutures	Successfully healed

*Note:* Intraop = intraoperatively. Postop = postoperatively.

In our opinion, prevention of such a complication is possible. Preoperative assessment of features like a high‐arched palate and a wide maxillary crest can be helpful in anticipating the adverse event and preventing it. These clinical features can be detected on a careful physical examination with or without a CT scan of the paranasal sinuses. The intraoperative technique in handling a wide and hard maxillary crest can be one of the preventive measures (e.g., using a powered instrument like a drill instead of the mallet with chisel/osteotome). Endoscopy can be used for a meticulous inspection of the nasal septal bone before resection. Intraoperative detection of hard palate injury may be optimal to do a simple repair.

## 4. Conclusion

Injury to the hard palate is one of the possible, although rare, complications of septoplasty. To avoid such an event, it is prudent to thoroughly examine the oral cavity prior to the procedure to catch important features such as a high‐arched palate. Intraoperatively, extra care should be taken when handling thick and wide maxillary crests. In the event that a hard palate injury does take place, early intraoperative detection and primary repair are recommended for better outcomes.

## Funding

No funding was received for this manuscript.

## Consent

No written consent has been obtained from the patients as there is no patient identifiable data included in this case report/series.

## Conflicts of Interest

The authors declare no conflicts of interest.

## Data Availability

There are no data to be shared.
